# Exposure to Microplastics in Biological Matrices and Neurodevelopmental Outcomes in Children: A Systematic Review

**DOI:** 10.3390/nano16100618

**Published:** 2026-05-18

**Authors:** Francesco Fabrizio Comisi, Andrea Maria Comisi, Elena Esposito, Vassilios Fanos

**Affiliations:** 1 Neonatal Intensive Care Unit, Department of Surgical Sciences, University of Cagliari, 09124 Cagliari, Italy; francesco.f.comisi@gmail.com (F.F.C.);; 2 Pediatric Unit, Department of Clinical and Experimental Medicine, University of Catania, 95131 Catania, Italy; comisiandrea@gmail.com

**Keywords:** pediatrics, cognition, child behavior, neurotoxicity, environmental exposure, biological monitoring, epidemiologic studies, GRADE

## Abstract

Micro- and nanoplastics (MNPs) are ubiquitous environmental contaminants detected in numerous human tissues, yet epidemiological evidence on MNPs exposure and neurodevelopmental outcomes in children has not been systematically evaluated. We aimed to systematically identify, appraise, and synthesize observational evidence on this association in children aged 0–18 years. Six databases were searched on 19 February 2026 following the Preferred Reporting Items for Systematic Reviews and Meta-Analyses (PRISMA) 2020 guidelines (PROSPERO: CRD420261328979). Risk of bias and certainty of evidence were assessed using Joanna Briggs Institute (JBI) checklists and the Grading of Recommendations, Assessment, Development and Evaluations (GRADE) framework. Three studies met the inclusion criteria (all published in 2025, China; *n* = 30–5670; two studies with probable population overlap), addressing behavioral, cognitive, and neurological outcome domains, encompassing 56 associations across 14 outcomes. Each study showed a uniform direction of association (higher MP exposure was associated with poorer outcomes); however, probable population overlap between Dong and Zheng precludes interpretation of this pattern as independent cross-study replication. All outcomes were rated Very Low certainty under GRADE; meta-analysis was not performed. Although experimental evidence supports biological plausibility, no causal inferences can be drawn in the absence of independent replication, and the field remains at the stage of hypothesis generation. Future studies should prioritize prospective longitudinal designs, spectroscopic exposure confirmation, and standardized neurodevelopmental outcomes.

## 1. Introduction

MNPs, defined as synthetic polymer particles less than 5 mm in diameter, are ubiquitous environmental contaminants of growing concern for human health. These particles have been detected in over 20 human tissues and biological fluids, including blood, placenta, lung tissue, breast milk, urine, and feces [1]. Children face disproportionately high exposure, with an estimated 3.3-fold greater dietary MNPs intake per kilogram of body weight than adults, driven by age-specific behaviors and contaminated sources, including baby food containers, which release millions of microplastic particles under heating conditions [2,3]. These factors have led to the identification of children as a priority population for MNPs health risk assessment [4]. Despite growing concern, the human epidemiological evidence on MNPs exposure and neurodevelopmental outcomes in children has not been systematically evaluated. Previous reviews have addressed adjacent questions: Symeonides et al. [5] conducted an umbrella review of 759 meta-analyses on plastic-associated chemicals such as phthalates and bisphenol A, but addressed chemical additives rather than MNPs particles, which involve distinct biological mechanisms and exposure assessment methods; Vojnits et al. [6] systematically reviewed MNPs neurotoxicity across 234 original research articles, finding extensive experimental evidence but no human epidemiological data; and Nadarasan et al. [7], in a scoping review of 45 studies on MNPs and children’s health, identified no human studies linking MNPs particles to neurodevelopment. To the best of the authors’ knowledge, no systematic review has evaluated this specific intersection of MNPs exposure, human epidemiological evidence, and pediatric neurodevelopmental outcomes. Experimental evidence nonetheless supports the biological plausibility of MNPs neurotoxicity. MNPs can cross the blood–brain barrier (BBB) and accumulate in brain tissue, with size-dependent translocation demonstrated in animal models [8]. MNPs have also been detected in human cerebrospinal fluid, with selective polymer accumulation [9], and a recent autopsy study in adult decedents reported that microplastics constituted approximately 0.5% of human brain tissue by weight, with concentrations 7- to 30-fold higher than in liver or kidney and a 50% increase between 2016 and 2024 samples; notably, individuals with dementia had substantially higher brain plastic concentrations [10]. Proposed neurotoxicity mechanisms include neuroinflammation, now recognized as a common pathogenic pathway across neurological disorders [11], with nuclear factor kappa-light-chain-enhancer of activated B cells (NF-κB)-mediated cytokine release, oxidative stress, and disruption of neuronal signaling [1]. Children are uniquely vulnerable to MNPs-related neurotoxicity owing to heightened exposure and biological susceptibility. Infant feeding bottles release millions of microplastic particles per use during formula preparation, and mouthing and crawling behaviors further amplify oral ingestion during early life [12]. The developing BBB is not fully mature, with tight junction protein expression at approximately 60% of adult levels in animal models [2], potentially facilitating even greater MNPs translocation and accumulation than observed in adult brain tissue [10] during critical windows of neuronal migration, synaptogenesis, and myelination. MNPs have been detected in pediatric biological samples [13], and prenatal exposure via placental transfer has been documented, with microplastics detected in human placentas and associated with adverse birth outcomes [14,15,16,17]. Prenatal MNPs exposure has also been shown to affect fetal neurodevelopment in animal models [18]. The established disproportionate neurodevelopmental burden of environmental toxicants on children [19,20] provides the rationale for investigating MNPs as a potential new exposure of concern. The objective was to systematically identify, appraise, and synthesize observational evidence on the association between MNPs exposure in human biological matrices and neurodevelopmental outcomes in children aged 0 to 18 years.

## 2. Materials and Methods

### 2.1. Protocol and Registration

This systematic review was retrospectively registered on PROSPERO (CRD420261328979). The review was reported following the PRISMA 2020 guidelines [21] and the Synthesis Without Meta-analysis (SWiM) reporting guideline for narrative synthesis [22]. No protocol deviations occurred. All pre-specified main outcome domains (behavioral, cognitive, neurological, psychological) were identified in the eligible studies, except motor and language development, which were not reported in any included study. Pre-specified additional outcomes, neuroimaging biomarkers (hippocampal brain iron) and circulating inflammatory biomarkers (interleukin-6 [IL-6]), were reported by Xie et al. [23] and are included in the synthesis.

### 2.2. Eligibility Criteria

Studies were eligible if they met the following PECOS criteria. Population: children and adolescents aged 0 to 18 years, or prenatal exposure with neurodevelopmental outcomes measured during childhood, with no restrictions on health status, geographic location, or clinical setting. Exposure: MNPs as physical particles measured in any human biological matrix (e.g., urine, feces, blood, placenta, meconium, or breast milk), using any analytical method. Comparator: for case–control studies, healthy age- and sex-matched controls; for cross-sectional studies, no formal comparator was required, with associations examined across exposure gradients. Outcomes: neurodevelopmental outcomes including behavioral problems, cognitive function, seizure disorders, anxiety, neuroimaging biomarkers, and motor or language development. Study design: cross-sectional, cohort (prospective or retrospective), case–control, case series, or ecological. Effect measures of interest included regression coefficients, odds ratios, correlation coefficients, and mean differences with 95% confidence intervals. Studies were excluded if they were reviews, editorials, commentaries, conference abstracts, in vitro or animal-only studies, or case reports. Studies measuring only chemical additives (e.g., phthalates, bisphenol A) without MNPs particle quantification, or reporting only environmental contamination without human biomonitoring, were also excluded.

### 2.3. Information Sources and Search Strategy

Six databases were searched on 19 February 2026: PubMed, Scopus, Cochrane Library (CENTRAL), Web of Science, ClinicalTrials.gov, and WHO ICTRP. No date restrictions were applied; only English-language publications were included. The search strategy combined three concept groups using Boolean operators: (1) microplastics and nanoplastics; (2) pediatric populations; and (3) neurodevelopmental outcomes. The complete search strings for all databases are provided in Supplementary File S1. Strategies were adapted to the syntax and controlled vocabulary of each database. The search identified 478 records. Forward citation searching and backward reference list checking of included studies yielded 105 additional records, none of which met the inclusion criteria.

### 2.4. Study Selection

Two reviewers (A.M.C., F.F.C.) independently screened titles and abstracts using a web-based systematic review management platform. Inter-rater reliability was almost perfect (Cohen’s kappa = 0.829; calculated across all 346 screened records). Seven disagreements were resolved by a third reviewer (E.E.) serving as arbiter. Full-text articles were subsequently assessed for eligibility independently by the same reviewers with the same resolution process; no disagreements arose at this stage. Reasons for full-text exclusion were recorded for each excluded article.

### 2.5. Data Extraction

Two reviewers (A.M.C., F.F.C.) independently extracted data using a standardized form developed a priori. Discrepancies were resolved by consensus with third-reviewer arbitration (E.E.). The corresponding author of Xie et al. [23] was contacted during the data extraction phase and provided supplementary data, including mean differences with 95% confidence intervals for key group comparisons and clarification of the confounder control procedures used at participant selection (age, sex, socioeconomic status, mental and endocrine disorders, and other environmental exposures; dietary habits were not adjusted); these clarifications are reflected in the risk-of-bias narrative in Section 3.3 and in the limitations section. No further author re-contact was pursued because the available clarifications had been obtained; the corresponding authors of the other two included studies were also contacted but did not respond.

### 2.6. Risk of Bias Assessment

Risk of bias was assessed using two JBI Critical Appraisal Checklists: the Checklist for Analytical Cross-Sectional Studies (8 items) [24] for Dong et al. [25] and Zheng et al. [26], and the Checklist for Case-Control Studies (10 items) [24] for Xie et al. [23]. Inter-reviewer agreement was almost perfect (Cohen’s kappa = 0.843, raw agreement 92.3%) across 26 domain-level assessments.

### 2.7. Certainty of Evidence

Certainty was assessed using the GRADE approach [27], starting at Low for observational evidence. Five downgrading and three upgrading domains were evaluated for each exposure–outcome pair.

### 2.8. Data Synthesis

A narrative synthesis was adopted, reported following the SWiM guideline [22]; item-by-item adherence is documented in Supplementary File S1. Meta-analysis was not performed owing to heterogeneous outcome domains across studies and insufficient independent studies for meaningful pooled estimates.

## 3. Results

The results are organized by individual study, as no outcome was assessed by more than one independent study.

### 3.1. Study Selection

**Figure 1:**
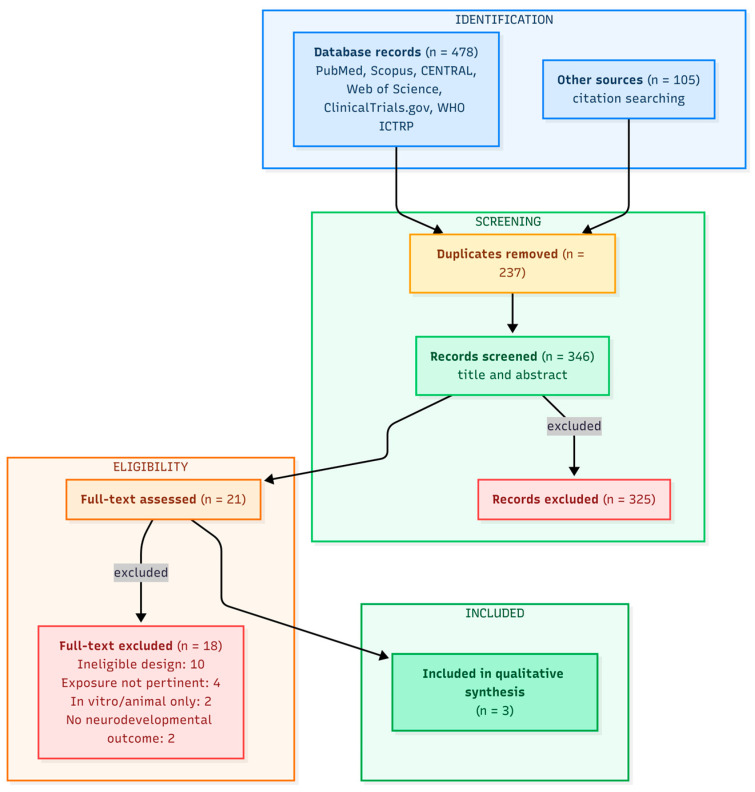
PRISMA 2020 flow diagram of the study selection process.

A total of 478 records were identified from databases. After the removal of 237 duplicates, 241 unique records remained. An additional 105 records from citation searching were screened alongside the database records, yielding 346 records screened in total. Of these, 325 were excluded at title and abstract screening. Of the 21 full-text articles assessed, 18 were excluded for the following reasons: ineligible study design (*n* = 10), exposure not pertinent (*n* = 4), in vitro/animal only (*n* = 2), and no neurodevelopmental outcome (*n* = 2); individual citations with exclusion reasons are listed in Supplementary File S1. Three studies met all the inclusion criteria [23,25,26]. The selection process is summarized in Figure 1.

### 3.2. Study Characteristics

None of the included studies specifically measured nanoplastic particles; accordingly, “MPs” (microplastics) is used hereafter when referring to measured exposures. All three studies were published in 2025, conducted in China, and addressed distinct neurodevelopmental outcome domains (Table 1). Dong et al. [25] and Zheng et al. [26] share probable population overlap: both were conducted in Shenyang by researchers affiliated with China Medical University during 2022–2023, recruited from 40 primary schools and using identical analytical methods (optical microscopy, Carl Zeiss Axio-Lab A1; Carl Zeiss Microscopy GmbH, Jena, Germany) to quantify polyamide (PA), polypropylene (PP), and polyvinyl chloride (PVC) in urine. The Dong sample (*n* = 1000, age 6–9 years) largely overlaps with the Zheng cohort (*n* = 5670, age 7–10 years), although the age ranges are not fully congruent (Dong includes 6-year-olds not covered by Zheng), indicating that Dong probably represents a partial subsample. Both studies used scanning electron microscopy with energy-dispersive X-ray spectroscopy (SEM-EDS) for compositional confirmation on sample subsets, with Dong additionally applying micro-Raman spectroscopy. The degree of population overlap remains unconfirmed. The studies covered complementary outcome domains: Zheng assessed cognitive function through n-back working memory and Attention Network Test tasks, while Dong assessed behavioral problems using the parent-reported Strengths and Difficulties Questionnaire (SDQ). Xie et al. [23] reported a multi-component study comprising an adult occupational cohort, a pediatric cohort, and a mouse model; only the pediatric cohort met this review’s inclusion criteria. This component was classified as case–control because participants were selected on the basis of disease status (newly diagnosed epilepsy vs. healthy controls) rather than exposure, comparing 15 children with newly diagnosed epilepsy and 15 age- and sex-matched healthy controls recruited from a single hospital site (Affiliated Hospital of Jiangnan University) in Wuxi, Jiangsu Province. Fecal MPs (polyethylene [PE], PP, polystyrene [PS]) were quantified using Raman spectroscopy, providing chemical confirmation of polymer identity. This study was the only one to contribute neurological outcome data, including seizure severity, anxiety (Generalized Anxiety Disorder 7-item scale [GAD-7]), and hippocampal brain iron levels measured by T2-weighted magnetic resonance imaging (MRI). Regarding funding, Zheng and Dong did not report funding sources; Xie was supported by the National Natural Science Foundation of China and other grants.

### 3.3. Risk of Bias Assessment

Risk of bias results are presented in Supplementary Tables S1 and S2 (Supplementary File S1). Zheng et al. [26] and Dong et al. [25] shared a similar risk-of-bias profile, with favorable ratings on most cross-sectional checklist domains. The primary concern was exposure measurement (Domain 3, rated No): both studies relied on optical microscopy for urinary MP quantification. SEM-EDS and micro-Raman spectroscopy were applied to sample subsets for compositional confirmation, but systematic spectroscopic verification of all counted particles was not performed. For Zheng, statistical analysis (Domain 8) was rated Unclear because multiple analytical approaches (quantile g-computation vs. generalized quantile-weighted sum [gQWS]) yielded discrepant results for certain associations. Xie et al. [23], assessed with the JBI case–control checklist, received favorable ratings on four of ten items. Principal limitations included the absence of multivariable confounding adjustment in the published analyses, although author correspondence clarified that groups were controlled at participant selection for age, sex, socioeconomic status, and key comorbidities; dietary habits were not controlled. Additional concerns included a single-timepoint fecal exposure measure, no multiple comparison correction, and bivariate analyses in a sample of only 30 participants. A relative strength was the use of Raman spectroscopy for chemical polymer confirmation.

### 3.4. Individual Study Results

The three studies examined 56 exposure–outcome associations: 18 in Zheng, 28 adjusted in Dong, and 10 crude in Xie. All statistical details are presented in Table 2, Table 3 and Table 4; the narrative below describes patterns and directions.

#### 3.4.1. Zheng et al. [26]

Zheng et al. [26] examined associations between urinary MP concentrations (PA, PP, PVC, total MPs) and three cognitive outcomes in 5670 children using linear mixed models adjusted for age, body mass index (BMI), sex, parental education, family income, environmental tobacco smoke exposure, and breastfeeding history.

Higher MP concentrations were negatively associated with working memory performance (Two-Back d’). PA showed the strongest association, followed by total MPs and PP; PVC was not statistically significant. For superior working memory (Three-Back d’), PVC, PP, and total MPs were significantly associated with poorer performance, while PA was marginally non-significant. Mixture analyses using quantile g-computation confirmed significant negative associations for both working memory outcomes, while gQWS models did not reach significance for any outcome, indicating that mixture effect conclusions are method-dependent (Table 2). The association between total MPs and inattentiveness (hit reaction time standard error [HRT-SE]) was paradoxically negative, indicating less inattentive performance with higher exposure. This unexpected direction, combined with marginal significance and inconsistency across analytical methods, suggests it may represent a chance finding; possible explanations are discussed in Section 4.5.

#### 3.4.2. Dong et al. [25]

Dong et al. [25] assessed associations between urinary MP concentrations and seven SDQ behavioral outcomes in 1000 children using mixed-effect negative binomial regression, adjusted for BMI, age, parental education, household income, sex, environmental tobacco smoke, and breastfeeding status. A consistent pattern of positive associations was observed between MP concentrations and behavioral problem scores: of 28 adjusted associations (four polymer exposures crossed with seven SDQ outcomes, the four difficulty subscales, the prosocial subscale, the total difficulties score, and the impact supplement), 23 were statistically significant; the five non-significant tests were PVC × emotional problems and the four polymer × impact-supplement comparisons. All four MP types (total, PA, PP, PVC) were positively associated with conduct problems, hyperactivity–inattention, and peer relationship problems, and negatively associated with prosocial behavior (Table 3). PP showed the strongest per-polymer associations for most subscales. All four MP types were positively associated with the total difficulties score. None of the four MP types was significantly associated with the impact supplement, indicating that elevated behavioral scores did not translate into perceived functional impact. Crude analyses showed the same pattern, indicating that confounder adjustment did not materially alter findings. This 23-of-28 pattern reflects a single coherent within-study association (higher MP associated with poorer behavior) rather than 28 independent biological signals; polymer-specific or subscale-specific effects cannot be claimed (see Section 4.5.2 for the analytical caveats: SDQ inter-outcome correlation, polymer co-correlation, absence of multiple-testing correction, and parent-rater bias).

#### 3.4.3. Xie et al. [23]

Xie et al. [23] compared fecal MP concentrations between 15 children with epilepsy and 15 controls using unadjusted comparisons. Total MPs and all individual polymers (PE, PP, PS) were significantly higher in the seizure group, with large effect sizes for the key group comparisons (Table 4). When stratified by seizure severity based on duration (mild [<2 min] (*n* = 4), moderate [2–5 min] (*n* = 6), severe [≥5 min] (*n* = 5)), higher MP levels were observed with increasing severity; the corresponding author confirmed that a formal trend test was not feasible because the control group did not experience seizures. Blood IL-6 was significantly elevated in cases and positively correlated with fecal total MPs (Spearman R = 0.52; 95% CI: 0.20 to 0.74). The GAD-7 comparison yielded the largest effect size among Xie outcomes (d = 2.32; Table 4), although this instrument lacks established pediatric validation and the effect size should be interpreted considering the known anxiety comorbidity of pediatric epilepsy. See Section 4.5.3 for the full GAD-7 caveat (off-label pediatric use, parental-assisted administration, author-confirmed cross-cohort comparability rationale, and pediatric epilepsy comorbidity inflation per Scott et al. [28]). T2-weighted MRI revealed significantly elevated hippocampal brain iron levels in children with seizures compared with controls (Table 4). All associations are unadjusted, and the cross-sectional case–control design with disease-defined groups precludes inference about the direction of association (see Section 4.5.2 for explicit discussion of reverse causality); methodological considerations are detailed in Section 3.3 and reflected in the GRADE assessment (Section 3.5).

### 3.5. Certainty of Evidence

All 14 GRADE-assessed outcomes were rated Very Low certainty (Tables S3 and S4 of Supplementary File S1). This rating is consistent with the earliest stage of an evidence domain in which only observational designs are available, and no outcome has yet been independently replicated. Two distinct downgrade patterns emerged. For the 10 Dong and Zheng outcomes, certainty was downgraded one level for serious risk of bias (reliance on optical microscopy without systematic spectroscopic verification) and one level for serious indirectness (uncertain validity of urinary MPs as a biomarker of systemic MPs burden). Inconsistency could not be assessed (single-study evidence per outcome). Imprecision was not downgraded given adequate sample sizes with available confidence intervals. Potential upgrading for dose–response was considered based on Zheng’s Bayesian Kernel Machine Regression (BKMR) analyses, which showed negative dose-dependent associations between the MP mixture and working memory performance, but was judged insufficient for upgrading given the single-study, cross-sectional design and the reanalysis of the same data. For the four Xie outcomes, certainty was downgraded more severely: two levels for very serious risk of bias, two levels for very serious imprecision (*n* = 30), and one to two levels for indirectness, with GAD-7 anxiety receiving the most severe downgrade owing to the additional concern of using an adult-validated instrument in children (see Section 4.5.3 for full caveat). Regarding selective outcome reporting, Dong et al. reported all seven pre-specified SDQ subscales across four polymer exposures (28 adjusted associations); none of the impact supplement associations reached significance. Crude analyses were concordant and are not tabulated. Zheng et al. reported all three cognitive outcomes across four individual polymers and two mixture methods (18 associations). Xie et al. reported all pre-specified neurological outcomes (10 unadjusted comparisons). Publication bias could not be formally assessed owing to the small number of included studies.

## 4. Discussion

### 4.1. Summary and Comparison with Existing Literature

This review identified three studies addressing the association between MP exposure measured in biological matrices and neurodevelopmental outcomes in children. All 14 outcomes were rated Very Low certainty under GRADE, with the explicit caveat that no inferential claims should be drawn beyond hypothesis generation. Several findings nonetheless merit cautious consideration. The two Shenyang cross-sectional studies (probable population overlap; see Section 3.2) reported uniform-direction associations between urinary MPs and behavioral and cognitive outcomes: Zheng et al. [26], with 5670 participants, found uniform-direction negative associations between urinary MPs and working memory performance, while Dong et al. [25], with 1000 participants, reported uniform-direction associations across 23 of 28 adjusted exposure–outcome combinations spanning four polymer types and seven SDQ outcomes, a pattern maintained after confounder adjustment. Because the two studies share probable population overlap, their agreement cannot be considered independent replication; we therefore interpret this body of evidence as a single Shenyang signal rather than as two converging studies. Xie et al. [23], despite its small sample (*n* = 30), provides the only case–control data and the only neuroimaging biomarker data in the included evidence base; however, the case–control design with disease-defined groups precludes any causal interpretation, and reverse causality (epilepsy altering fecal MP) is plausible alongside the forward direction (Section 4.5.2). The studies addressed complementary outcome domains—behavioral, cognitive, and neurological—collectively covering a broad spectrum of neurodevelopmental endpoints, though no single outcome was examined by two independent studies. The publication of three studies in a single year (2025) signals rapidly growing research interest in this topic. A nuance within the Shenyang behavioral data warrants brief comment. In Dong et al. [25], higher MP exposure correlated with all six SDQ symptom-level outcomes (the four difficulty subscales, prosocial behavior, and total difficulties; 23 of 24 tests significant)—trait-level ratings of the child’s general behavioral functioning—but not with the SDQ impact supplement, a single composite score in which parents rate whether those difficulties cause distress or impairment across daily life contexts (home, friendships, classroom learning, leisure activities; 0 of 4 polymer × impact tests significant). The pattern indicates that higher MP exposure tracks more parent-reported symptoms, but not greater parent-perceived functional impairment. Several factors are consistent with this dissociation. The adjusted symptom-level magnitudes (beta approximately 0.13 to 0.23 per IQR increase, log rate ratios) may register at item-level ratings without cumulatively crossing the threshold for perceived impairment. The impact supplement is psychometrically constructed as a dimension distinct from symptom presence, so divergence between them reflects validated construct separation rather than analytical inconsistency. A uniform parent-rater halo would inflate impact and symptom ratings concordantly; its absence on impact argues against that account, without excluding more selective forms of rater bias. The symptom-level associations have therefore not been shown to translate into caregiver-observable functional consequences at the exposure ranges studied, a distinction relevant for clinical and public-health framing (see Section 4.6). Across the three included studies, the single shared feature is a uniform direction of association at the within-study level: higher MP exposure aligns with poorer behavioral, cognitive, or neurological outcomes. In every other respect, the studies are heterogeneous, and that heterogeneity constrains what an integrative reading can support. The matrices differ (urinary MPs in the Shenyang cohort, fecal MPs in Xie et al. [23]) and so do the polymer panels (PA/PP/PVC vs. PE/PP/PS), the age ranges (6–9 vs. 7–10 vs. ~9 years), the design (cross-sectional vs. case–control), the analytical confirmation depth (optical microscopy with subset SEM-EDS vs. Raman with field-emission SEM), and the confounder-adjustment sets. Effect-size variation across these studies is therefore expected on theoretical grounds: urinary MPs index a size-restricted, partly soluble particle fraction filterable through the renal route, whereas fecal MPs index unabsorbed luminal particles plus biliary excretion of an absorbed fraction, and blood, the only matrix that genuinely indexes systemic exposure [29], was not measured by any included study. The directional uniformity is therefore best read as qualitative consistency of sign, not quantitative consistency of magnitude. The present evidence cannot adjudicate cross-domain versus domain-specific MP effects, matrix-driven effect-size variation, or polymer-specific signals; Table 5 summarizes the three studies on these axes. These distinctions become tractable only when independent studies become available with comparable matrices, polymer panels, and outcome instruments, ideally with prospective designs that anchor the temporal sequence between exposure and outcome. These patterns are insufficient to establish causation. Nonetheless, they provide an informative foundation for targeted future investigation. As documented by Yaffe et al. [30], systematic reviews yielding few included studies serve a recognized function in establishing evidence baselines and identifying research priorities. This review is, to the best of our knowledge, the first to bridge MPs exposure in biological matrices, human epidemiological evidence, and pediatric neurodevelopmental outcomes, three dimensions previously addressed separately. Symeonides et al. [5] examined plastic-associated chemicals rather than particles; Vojnits et al. [6] reviewed experimental MNPs neurotoxicity across 234 studies without finding human data; Nadarasan et al. [7] confirmed the absence of human MNPs-neurodevelopment studies in their scoping review; and Makwana et al. [31] used ecological rather than individual-level exposure data in adult populations.

### 4.2. Environmental Analogy

The trajectory of evidence development for established environmental neurotoxicants provides a useful methodological context. The Faroe Islands methylmercury birth cohort required decades of prospective follow-up and independent replication in New Zealand and Seychelles cohorts to establish dose–response relationships between prenatal exposure and cognitive deficits [32]. Similarly, the association between organophosphate pesticide exposure and attention-deficit/hyperactivity disorder risk was established through multiple independent birth cohorts across diverse populations [33]. By comparison, Symeonides et al. [5] documented 759 meta-analyses for plastic-associated chemicals, reflecting decades of accumulated research. The MPs evidence base, with three studies and no meta-analysis, is at the very beginning of this trajectory. The availability of studies with sample sizes exceeding 5000 participants within the field’s first year of publication is noteworthy relative to the early trajectories of most environmental neurotoxicant fields, and children’s heightened vulnerability to environmental neurotoxicants is well established for these legacy pollutants [19,32], providing the biological rationale for prioritizing MNPs research. We invoke this analogy to illustrate the methodological trajectory required to establish a new neurotoxicant, not to imply that MPs have already been established as such.

### 4.3. Biological Plausibility

The included evidence is interpreted under an explicit working hypothesis: MPs translocate from peripheral routes (gastrointestinal, respiratory) into systemic circulation, cross the blood–brain barrier, deposit in brain parenchyma, and trigger neuroinflammation, oxidative stress, and ferroptotic cell death, pathways shared with established environmental neurotoxicants [32]. The BBB’s tight-junction integrity is incomplete in animal models of the perinatal and early-childhood windows [2], although whether a comparable permeability exists in human children remains an extrapolation requiring direct testing. This hypothesis encompasses three nested forms: a particle-mediated effect (mechanical injury, membrane interaction), a chemical-mediated effect (additives intrinsic to particles and chemicals adsorbed during environmental transit), and the most plausible mixed form combining both. The human epidemiological evidence reviewed here cannot distinguish between these contributions and remains to be tested by prospective epidemiological studies that co-measure MP particles and plastic-associated chemicals. Biological plausibility is necessary but not sufficient for causal inference; the human evidence base remains at Very Low GRADE certainty for every outcome. Substantial experimental evidence from animal models and in vitro systems supports the biological plausibility of MPs neurotoxicity. In animal models, MPs cross the BBB with size-dependent translocation, with particles smaller than 500 nm crossing more readily and accumulating in brain parenchyma [8]. Direct evidence of central nervous system penetration in humans comes from a small case series in which MPs were detected in human cerebrospinal fluid samples, with polyethylene and polyvinyl chloride being the predominant polymers identified [9]. Mechanisms documented in animal models and in vitro systems include NF-κB-mediated neuroinflammation with elevated IL-6 and TNF-alpha [1], relevant to the IL-6 elevation correlated with fecal MPs in Xie et al. [23], and oxidative stress via reactive oxygen species generation and lipid peroxidation in neural tissue. Developing neurons are particularly vulnerable to oxidative damage owing to their high metabolic rate and immature antioxidant defenses [19]. The mouse model component of Xie et al. [23] demonstrated hippocampal ferroptosis via glutathione peroxidase 4 downregulation, with rescue by ferrostatin-1, providing a mechanistic framework consistent with the elevated hippocampal iron observed in the pediatric cohort. Post-mortem analyses of adult human brain tissue [10] reported that microplastics constituted approximately 0.5% of brain tissue by weight, with concentrations 7- to 30-fold higher than in liver or kidney; this establishes that translocation occurs in adults but does not establish dose–response between any peripheral matrix and CNS burden. In animal models, the immature BBB, with zonula occludens-1 expression at approximately 60% of adult levels [2], may facilitate greater MPs translocation during critical developmental windows of neuronal migration, synaptogenesis, and myelination. Additional pathways include gut–brain axis disruption [13] and endocrine effects [12], though neither was directly assessed in the included studies. What each biological matrix sampled in the included studies represents physiologically determines what MP measurements can and cannot reveal about brain-relevant exposure. Urinary MPs reflect renal and lower urinary tract excretion; fecal MPs reflect transit of unabsorbed luminal particles plus biliary excretion of an absorbed fraction [34,35]. Blood is the only peripheral matrix that genuinely indexes systemic exposure [29]; none of the included studies measured blood. None of the matrices used in the included studies has been validated against MP deposition in brain or central nervous system tissue in humans, and a recent paired-matrix study confirmed that urine and feces capture different physiological compartments [36]. Available evidence on adult brain bioaccumulation [10] and organ-specific heterogeneity in post-mortem human tissues [37] establishes that translocation occurs but does not establish a dose–response relationship between any peripheral matrix and central nervous system burden. Perinatal exposure biomarkers (meconium, placenta, breast milk) index exposure during critical developmental windows; none of the three included studies sampled these matrices, although the broader human MP biomonitoring literature has demonstrated detectable particles in all of them [14,15,16,17]. The methodological state of MP biomonitoring has been characterized as preclusive of adequate risk assessment [38]; the within-study associations reported by the included studies should be interpreted under this constraint.

### 4.4. Strengths

This review adhered to PRISMA 2020 and SWiM reporting guidelines and was registered on PROSPERO (CRD420261328979). All key steps were performed independently by two reviewers with high inter-rater reliability: screening (Cohen’s kappa = 0.829, almost perfect agreement), data extraction (discrepancies resolved by consensus with third-reviewer arbitration), and risk of bias assessment (Cohen’s kappa = 0.843, 92.3% raw agreement). GRADE was applied systematically to all 14 exposure–outcome pairs with evidence profile tables. The comprehensive search encompassed six databases plus forward and backward citation searching (105 additional records screened), providing confidence that the evidence base was captured. Documenting this early evidence base serves the recognized function of “near-empty” systematic reviews in establishing baselines and directing research [30].

### 4.5. Limitations

This review has important limitations, organized below by the methodological axis on which they bear. Only three studies met the inclusion criteria, two with probable population overlap, and all 14 outcomes were rated Very Low GRADE certainty.

#### 4.5.1. Exposure Assessment

Exposure assessment in the three included studies has four interlocking limitations. MPs were measured at a single timepoint, which cannot capture cumulative exposure during the prenatal and early-childhood windows that environmental neurotoxicology identifies as most relevant to neurodevelopmental outcomes. The biological matrices used (urine, feces) are indirect proxies of brain-relevant exposure: none has been validated against MP deposition in brain or central nervous system tissue in humans (see Section 4.3 for matrix-specific physiology). Optical microscopy is the dominant identification method in Zheng et al. [26] and Dong et al. [25]; visual identification of MPs without systematic spectroscopic confirmation is known to introduce both false-positive and false-negative errors, particularly for small particles, and chemical confirmation by Fourier-transform infrared or Raman spectroscopy is therefore strongly recommended for unambiguous identification [39]. Spectroscopic confirmation by SEM-EDS or micro-Raman was applied to subsets only, with confirmation rates not reported. The dominant misclassification pattern is plausibly non-differential, attenuating association magnitudes toward the null, but differential misclassification cannot be excluded if visual identification is density-dependent or correlated with sample matrix density (e.g., dietary fiber intake differs by socioeconomic status). Finally, MPs were measured in isolation across the three included studies; plastic-associated chemicals such as phthalates, bisphenol A, and per- and polyfluoroalkyl substances are well-documented neurotoxicants and endocrine disruptors [5] but were not measured alongside MPs, so the observed associations cannot be attributed to particles independently of this chemical cargo. This indirectness drove the GRADE downgrade for indirectness already applied in Section 3.5.

#### 4.5.2. Confounding and Causal Inference

Beyond the confounders adjusted for in Dong et al. [25] and Zheng et al. [26] (sex, age, BMI, parental education, family income, environmental tobacco smoke, breastfeeding), several established or plausible confounders were not addressed. Dietary patterns are the dominant pathway of pediatric MP exposure [4] and co-exposure to plastic-associated chemicals (BPA, phthalates, PFAS), themselves established neurodevelopmental toxicants and endocrine disruptors reviewed in detail by Symeonides et al. [5], was not measured in any of the three included studies; the observed associations cannot therefore be attributed to particles independently of this chemical cargo. For Xie et al. [23], case–control matching at recruitment does not preclude residual confounding from medication, diet, hospital-environment factors, and dysbiosis specific to children with newly diagnosed epilepsy. A separate analytical concern specific to Dong et al. [25] is that the 23-of-28 significance pattern reflects a single coherent within-study association rather than independent biological signals. The pattern is jointly produced by four statistical features: the seven SDQ outcomes are positively inter-correlated by construction [40]; the four polymer exposures are intra-individually correlated within urinary samples; no multiple-testing correction was applied across the 28 association tests; and parent-rater bias may inflate apparent uniformity. Polymer-specific or subscale-specific effects therefore cannot be claimed. Two threats to causal inference apply. First, in Xie et al. [23], reverse causality is plausible alongside the forward direction [41]: epilepsy and its treatment can alter fecal MP load via altered diet, antiepileptic drug formulations whose plastic packaging contributes to MP intake, gastrointestinal motility alterations, and overrepresentation in hospital-care environments. We retain the Xie et al. [23] data because they provide the only case–control design, the only neuroimaging biomarker data, the only inflammatory biomarker, and the only quantitative anxiety data in the eligible literature, while explicitly downgrading its causal interpretation. Second, point-in-time MP measurement (urinary MPs index hours–days of systemic exposure, fecal MPs 24–72 h of gastrointestinal transit) cannot capture cumulative exposure across the prenatal and early-childhood windows most relevant to neurodevelopment [32]; steady-state assumptions linking point measurement to chronic exposure remain untested in pediatric populations and should be addressed by repeated-measures biomonitoring in prospective cohorts (e.g., the Ma’anshan Birth Cohort) [42].

#### 4.5.3. Outcome Assessment

Outcome assessment is heterogeneous across the three included studies: no instrument is shared by more than one study, precluding cross-study comparison at the outcome level. The most severe outcome-assessment limitation applies to the GAD-7 in Xie et al. [23]. The GAD-7 was applied off-label: published pediatric validations cover adolescents from approximately 11 y, with none extending to the school-age children of the Xie et al. [23] sample (mean ~9 y); the instrument was administered with parental assistance, introducing a separate validity concern; and the corresponding author confirmed in correspondence that the GAD-7 was selected to maintain direct comparability of anxiety scores with the adult occupational cohort (Cohort 1) of the same study, with no formal pediatric reliability or validity assessment performed in the children’s cohort. The observed Cohen’s d = 2.32 is also consistent with the documented anxiety comorbidity of pediatric epilepsy at diagnosis (pooled prevalence 18.9%, d ≈ 0.57 vs. healthy controls) [28], suggesting the magnitude is plausibly inflated by comorbidity rather than reflecting a quantitative MP-specific effect. This was the basis for the most severe GRADE downgrade applied to the GAD-7 outcome (Section 3.5).

#### 4.5.4. Generalizability and Reporting

Generalizability is constrained on two axes. Dong et al.’s [25] and Zheng et al.’s [26] probable populations overlap, so any apparent agreement between them cannot constitute independent replication; as a narrative sensitivity reading, restricting the synthesis to the two studies without overlap yields evidence on behavioral and neurological outcomes only, with no cross-study domain overlap, and the cognitive findings become uncorroborated. This restricted reading is even less informative than the primary synthesis and strengthens the call for independent replication outside Shenyang. All three included studies were conducted in urban China. The search was English-only, and publication bias could not be formally assessed owing to the small number of included studies.

### 4.6. Implications

#### 4.6.1. Implications for Research

The highest priority is establishing prospective birth or childhood cohorts with repeated MNPs exposure measurements and neurodevelopmental follow-up, as current cross-sectional designs cannot establish a temporal sequence. Future studies should employ Raman or Fourier-transform infrared spectroscopy for systematic polymer confirmation in all quantified particles [43], use validated pediatric neurodevelopmental instruments with established norms to enable cross-study comparison, and, for anxiety assessment specifically, employ pediatric-validated instruments such as the Screen for Child Anxiety Related Disorders (SCARED) or the Revised Children’s Anxiety and Depression Scale (RCADS), consistent with the International Consortium for Health Outcomes Measurement (ICHOM) consensus on a standard set of patient-reported outcome measures for child and youth anxiety, depression, OCD and PTSD [44], rather than adult-validated scales applied off-label; more broadly, comprehensive clinically normed batteries (e.g., Wechsler Intelligence Scale for Children [WISC], Child Behavior Checklist [CBCL]) should be considered alongside experimental paradigms to facilitate cross-study comparison, include adequate sample sizes with formal power analyses and adjustment for established confounders of child neurodevelopment, and consider multi-matrix exposure assessment (urine, feces, blood) within the same participants to understand relationships between different exposure metrics. The Ma’anshan birth cohort [42], with planned MP exposure assessment alongside neurodevelopmental outcomes, represents a model study design addressing several limitations identified here. Future studies should also account for age-specific exposure pathways, including mouthing behaviors and pediatric-specific contamination sources [4]. Independent replication of findings in populations outside China is essential to establish generalizability.

#### 4.6.2. Implications for Clinical Practice

Current evidence does not support specific clinical recommendations regarding MNPs exposure and neurodevelopmental risk. Nonetheless, given the well-characterized pediatric exposure pathways, including dietary intake, inhalation, and dermal contact [13], general precautionary measures are consistent with broader environmental-health hygiene practices, although they are not specifically supported by the very low-certainty MP-specific evidence reviewed here: avoiding heating food in plastic containers, preferring glass or stainless-steel feeding bottles, choosing natural-fiber clothing, and reducing children’s exposure to synthetic materials during early childhood. These measures carry no known risk and align with broader environmental health recommendations. As the evidence base matures through methodologically robust prospective studies, more targeted clinical guidance for the protection of the pediatric population is anticipated.

#### 4.6.3. Implications for Policy

A tension exists between the precautionary principle, given strong mechanistic plausibility, and the limited human epidemiological evidence currently available. These findings do not support specific policy interventions but underscore the need for research funding prioritizing human epidemiological studies in pediatric populations, within the broader context of plastic pollution as a systemic environmental challenge with potential implications across multiple dimensions of children’s health [45].

## 5. Conclusions

This systematic review provides the first comprehensive evaluation of human epidemiological evidence on MPs exposure and neurodevelopmental outcomes in children. Three studies, all published in 2025 and conducted in China, addressed behavioral, cognitive, and neurological outcome domains, although two studies share probable population overlap. All 14 outcomes were rated Very Low certainty under GRADE, no outcome has been independently replicated, and no causal inferences can be drawn. Despite these limitations, which are inherent to the earliest stage of a new evidence domain, the within-study uniformity of association direction, the substantial sample size of the largest cross-sectional cohort (*n* = 5670), though offset by the very small case–control sample (*n* = 30), and the strong mechanistic plausibility from experimental models collectively suggest, at the level of hypothesis generation rather than evidentiary support, that this evidence domain warrants sustained investigation. The rapid pace of publication in 2025 suggests the field is poised for substantial growth. Advancing it will require prospective longitudinal designs, spectroscopic exposure confirmation, standardized neurodevelopmental outcomes, adequate sample sizes with confounder adjustment, and multi-matrix exposure assessment to build the evidence base necessary for informed clinical and policy decisions regarding children’s MPs exposure and neurodevelopmental health. As this evidence base grows, precautionary measures to reduce children’s everyday exposure to plastic materials remain a prudent approach, carrying no known risk while the field works toward the robust evidence needed to inform targeted pediatric recommendations.

## Figures and Tables

**Table 1:** Characteristics of included studies examining the association between microplastic exposure in biological matrices and neurodevelopmental outcomes in children.

Author	Study Design	*n*	Age	Location	Biological Matrix	Analytical Method	Outcome Measure
Zheng et al. [26]	Cross-sectional	5670	7–10 years (median 9, IQR 2)	Shenyang, Liaoning Province, China	Urine	Optical microscopy (Carl Zeiss Axio-Lab A1, 100×; Carl Zeiss Microscopy GmbH, Jena, Germany); SEM-EDS	n-back task, ANT (cognitive)
Dong et al. [25]	Cross-sectional	1000	6–9 years (mean 8.5, SD 1.1)	Shenyang, Liaoning Province, China	Urine	Optical microscopy (Carl Zeiss Axio-Lab A1, 100×; Carl Zeiss Microscopy GmbH, Jena, Germany); SEM-EDS, micro-Raman	SDQ (behavioral)
Xie et al. [23]	Case–control	30	Mean 8.93 years (SD 1.83)	Wuxi, Jiangsu Province, China	Feces	Micro-Raman spectroscopy; FE-SEM	Seizure severity, GAD-7 (anxiety), brain iron (T2-MRI), blood IL-6

ANT, Attention Network Test; FE-SEM, field emission scanning electron microscopy; GAD-7, Generalized Anxiety Disorder 7-item scale; IQR, interquartile range; SD, standard deviation; SDQ, Strengths and Difficulties Questionnaire; SEM-EDS, scanning electron microscopy with energy-dispersive X-ray spectroscopy; T2-MRI, T2-weighted magnetic resonance imaging.

**Table 2:** Adjusted associations between urinary microplastic concentrations and cognitive outcomes in 5670 children aged 7–10 years (Zheng et al. [26]).

Exposure	Working Memory (Two-Back d’)	Superior Working Memory (Three-Back d’)	Inattentiveness (HRT-SE)
PA	−9.98 (−13.75 to −6.2; *p* < 0.001)	−1.4 (−2.84 to 0.05; *p* = 0.06)	−1.51 (−3.81 to 0.79; *p* = 0.20)
PP	−4.95 (−8.7 to −1.2; *p* = 0.01)	−1.77 (−3.2 to −0.34; *p* = 0.02)	−1.99 (−4.26 to 0.29; *p* = 0.09)
PVC	−2.28 (−6.08 to 1.52; *p* = 0.24)	−2.05 (−3.5 to −0.61; *p* = 0.01)	−0.74 (−3.04 to 1.57; *p* = 0.53)
Total MPs	−7.42 (−10.27 to −4.56; *p* < 0.001)	−2.25 (−3.34 to −1.16; *p* < 0.001)	−1.83 (−3.57 to −0.09; *p* = 0.04)
Mixture (g-comp)	−9.8 (−12.93 to −6.67; *p* < 0.01)	−2.35 (−3.55 to −1.16; *p* < 0.01)	−1.62 (−3.53 to 0.28; *p* = 0.09)
Mixture (gQWS)	−2.12 (−4.89 to 0.65; *p* = 0.13)	−0.88 (−1.8 to 0.03; *p* = 0.06)	−1.52 (−3.97 to 0.94; *p* = 0.22)

Beta coefficients (95% CI; *p*-value) per IQR increase from linear mixed models. d’, d-prime; g-comp, quantile g-computation; gQWS, generalized quantile-weighted sum; HRT-SE, hit reaction time standard error; MPs, microplastics; PA, polyamide; PP, polypropylene; PVC, polyvinyl chloride. Effect-size magnitudes should be interpreted in light of caveats detailed in Section 4.5.1 and Section 4.5.2.

**Table 3:** Adjusted associations between urinary microplastic concentrations and behavioral outcomes in 1000 children aged 6–9 years (Dong et al. [25]).

Exposure	Emotional	Conduct	Hyperactivity	Peer Problems	Prosocial	Total Difficulties	Impact
PA	0.157 (0.062 to 0.252; *p* = 0.001)	0.19 (0.098 to 0.282; *p* < 0.001)	0.192 (0.095 to 0.288; *p* < 0.001)	0.184 (0.084 to 0.284; *p* < 0.001)	−0.126 (−0.227 to −0.025; *p* = 0.014)	0.03 (0.023 to 0.037; *p* < 0.001)	−0.071 (−0.171 to 0.03; *p* = 0.168)
PP	0.157 (0.063 to 0.251; *p* = 0.001)	0.231 (0.14 to 0.322; *p* < 0.001)	0.18 (0.085 to 0.276; *p* < 0.001)	0.226 (0.128 to 0.324; *p* < 0.001)	−0.132 (−0.232 to −0.032; *p* = 0.010)	0.033 (0.026 to 0.04; *p* < 0.001)	−0.035 (−0.134 to 0.064; *p* = 0.489)
PVC	0.065 (−0.014 to 0.145; *p* = 0.106)	0.18 (0.104 to 0.257; *p* < 0.001)	0.12 (0.04 to 0.2; *p* = 0.003)	0.187 (0.102 to 0.271; *p* < 0.001)	−0.102 (−0.185 to −0.018; *p* = 0.018)	0.027 (0.02 to 0.035; *p* < 0.001)	0.031 (−0.053 to 0.114; *p* = 0.471)
Total MPs	0.128 (0.062 to 0.195; *p* < 0.001)	0.209 (0.146 to 0.273; *p* < 0.001)	0.168 (0.101 to 0.235; *p* < 0.001)	0.206 (0.137 to 0.275; *p* < 0.001)	−0.125 (−0.196 to −0.055; *p* = 0.001)	0.177 (0.148 to 0.206; *p* < 0.001)	−0.022 (−0.092 to 0.047; *p* = 0.528)

Beta coefficients (log rate ratios; 95% CI; *p*-value) per IQR increase from mixed-effect negative binomial regression. MPs, microplastics; PA, polyamide; PP, polypropylene; PVC, polyvinyl chloride. Effect-size magnitudes should be interpreted in light of caveats detailed in Section 3.4.2 and Section 4.5.2.

**Table 4:** Unadjusted associations between fecal microplastic concentrations and neurological outcomes in 30 children (15 with epilepsy, 15 controls; Xie et al. [23]).

Exposure	Outcome	Comparison	Effect	*p*
Total MPs (fecal)	Seizure group vs. controls	Group comparison	MD = 31.13 (95% CI: 14.35 to 47.91); d = 1.39	<0.01
PE (fecal)	Seizure group vs. controls	Group comparison	Higher in cases	<0.01
PP (fecal)	Seizure group vs. controls	Group comparison	Higher in cases	<0.01
PS (fecal)	Seizure group vs. controls	Group comparison	Higher in cases	<0.01
Total MPs (fecal)	Seizure severity (by duration)	Severity stratification	Higher with severity	NR
Seizure group	Blood IL-6	Group comparison	MD = 0.51 (95% CI: 0.05 to 0.97); d = 0.83	<0.05
Total MPs (fecal)	Blood IL-6	Spearman correlation	R = 0.52 (95% CI: 0.20 to 0.74)	<0.05
Seizure group	GAD-7 anxiety	Group comparison	MD = 6.73 (95% CI: 4.56 to 8.90); d = 2.32	<0.05
Total MPs (fecal)	GAD-7 anxiety	Higher MP group	Higher scores	<0.05
Seizure group	Hippocampal brain iron	Group comparison	Higher in cases	<0.05

All comparisons are unadjusted (Mann–Whitney U). MD and 95% CIs were obtained from the corresponding author; Cohen’s d was independently calculated assuming t-distribution (df = 28). Spearman R 95% CI estimated by Fisher’s z transformation (*n* = 30). CI, confidence interval; d, Cohen’s d; GAD-7, Generalized Anxiety Disorder 7-item scale; IL-6, interleukin-6; MD, mean difference; MPs, microplastics; NR, not reported; PE, polyethylene; PP, polypropylene; PS, polystyrene; R, Spearman correlation coefficient. Effect-size magnitudes should be interpreted in light of caveats detailed in Section 3.4.3, Section 4.5.2 and Section 4.5.3.

**Table 5:** Cross-study synthesis matrix of the three included studies.

Feature	Zheng et al. [26]	Dong et al. [25]	Xie et al. [23]
Design	Cross-sectional	Cross-sectional	Case–control
Population overlap	Probable overlap with Dong	Probable overlap with Zheng	Independent
Matrix	Urine	Urine	Feces
Polymer panel	PA, PP, PVC, total	PA, PP, PVC, total	PE, PP, PS, total
Sample size (n)	5670	1000	30 (15 cases + 15 controls)
Age (years)	7–10 (median 9)	6–9 (mean 8.5)	mean 8.93 (SD 1.83)
Outcome domain	Cognitive (working memory, attention)	Behavioral (SDQ, 7 subscales)	Neurological (seizures, GAD-7, IL-6, hippocampal iron)
Effect direction	Higher MPs → poorer working memory	Higher MPs → higher behavioral problems	Higher fecal MPs in cases (epilepsy)
Effect-size range	β −9.98 to −0.74 (per IQR)	β +0.027 to +0.231 (log rate ratios)	Cohen’s d 0.83–2.32; MD 0.51–31.13
Confounder adjustment	Age, BMI, sex, parental education, family income, ETS, breastfeeding	Same adjustment set as Zheng	None (matching at recruitment only)
Spectroscopic confirmation	SEM-EDS (subset only)	SEM-EDS + micro-Raman (subset only)	Raman + FE-SEM (per particle)
Risk of bias (JBI)	Domain 3 fail (optical microscopy); Domain 8 unclear	Domain 3 fail (optical microscopy)	4/10 domains favorable
GRADE certainty	Very Low (all 3 outcomes)	Very Low (all 7 outcomes)	Very Low (all 4 outcomes; GAD-7 most severely downgraded)

MPs, microplastics; PA, polyamide; PP, polypropylene; PVC, polyvinyl chloride; PE, polyethylene; PS, polystyrene; SD, standard deviation; SDQ, Strengths and Difficulties Questionnaire; SEM-EDS, scanning electron microscopy with energy-dispersive X-ray spectroscopy; FE-SEM, field-emission scanning electron microscopy; GAD-7, Generalized Anxiety Disorder 7-item scale; IL-6, interleukin-6; ETS, environmental tobacco smoke; BMI, body mass index; IQR, interquartile range; MD, mean difference; JBI, Joanna Briggs Institute.

## Data Availability

All data supporting the findings of this systematic review are contained within the article and Supplementary Materials. Mean differences with 95% confidence intervals presented in Table 4 were provided by the corresponding author of Xie et al. [23] following author correspondence; Cohen’s d values were independently calculated from the author-provided data.

## Supplementary Materials

The following supporting information can be downloaded at: https://www.mdpi.com/article/10.3390/nano16100618/s1, Complete search strategies for all databases, list of excluded full-text articles with reasons, SWiM reporting checklist, PRISMA 2020 checklist, risk of bias assessment tables (Tables S1 and S2), and GRADE certainty of evidence tables (Tables S3 and S4). References [21,22] are cited in the Supplementary Materials.

## References

[1] Dennis J., Arulraj D., Mistri T.K. (2025). Unseen Toxins: Exploring the Human Health Consequences of Micro and Nanoplastics. Toxicol. Rep..

[2] Luo Q., Tan H., Ye M., Jho E.H., Wang P., Iqbal B., Zhao X., Shi H., Lu H., Li G. (2025). Microplastics as an Emerging Threat to Human Health: An Overview of Potential Health Impacts. J. Environ. Manag..

[3] Hussain K.A., Romanova S., Okur I., Zhang D., Kuebler J., Huang X., Wang B., Fernandez-Ballester L., Lu Y., Schubert M. (2023). Assessing the Release of Microplastics and Nanoplastics from Plastic Containers and Reusable Food Pouches: Implications for Human Health. Environ. Sci. Technol..

[4] Lane T., Wardani I., Koelmans A.A. (2025). Exposure Scenarios for Human Health Risk Assessment of Nano- and Microplastic Particles. Micropl. Nanopl..

[5] Symeonides C., Aromataris E., Mulders Y., Dizon J., Stern C., Barker T.H., Whitehorn A., Pollock D., Marin T., Dunlop S. (2024). An Umbrella Review of Meta-Analyses Evaluating Associations between Human Health and Exposure to Major Classes of Plastic-Associated Chemicals. Ann. Glob. Health.

[6] Vojnits K., De León A., Gibon J., Barker P., Mahmoudi M., Pakpour S. (2025). A Systematic Review of the Potential Neurotoxicity of Micro-and Nanoplastics: The Known and Unknown. Part. Fibre Toxicol..

[7] Nadarasan S., Phuna Z.X., Zaman R., Tan C.K., Bustami N.A., Bin Ho Y., Kosasih S.J., Tan E.S.S. (2025). Microplastics and Child Health: A Scoping Review of Prenatal and Early-Life Exposure Routes and Potential Health Risks. Toxicol. Rep..

[8] Ragusa A., Fanos V. (2025). Microplastics and Nanoplastics in the Brain: A Review of the Neurodevelopmental Risks. J. Pediatr. Neonatal Individ. Med..

[9] Xie J., Ji J., Sun Y., Ma Y., Wu D., Zhang Z. (2024). Blood-Brain Barrier Damage Accelerates the Accumulation of Micro- and Nanoplastics in the Human Central Nervous System. J. Hazard. Mater..

[10] Nihart A.J., Garcia M.A., El Hayek E., Liu R., Olewine M., Kingston J.D., Castillo E.F., Gullapalli R.R., Howard T., Bleske B. (2025). Bioaccumulation of Microplastics in Decedent Human Brains. Nat. Med..

[11] Shi F.-D., Yong V.W. (2025). Neuroinflammation across Neurological Diseases. Science.

[12] Amran N.H., Zaid S.S.M., Mokhtar M.H., Manaf L.A., Othman S. (2022). Exposure to Microplastics during Early Developmental Stage: Review of Current Evidence. Toxics.

[13] Esposito E., Comisi F.F., Fanos V., Ragusa A. (2025). The Silent Conquest: The Journey of Micro- and Nanoplastics Through Children’s Organs. Toxics.

[14] Ragusa A., Svelato A., Santacroce C., Catalano P., Notarstefano V., Carnevali O., Papa F., Rongioletti M.C.A., Baiocco F., Draghi S. (2021). Plasticenta: First Evidence of Microplastics in Human Placenta. Environ. Int..

[15] Liu S., Guo J., Liu X., Yang R., Wang H., Sun Y., Chen B., Dong R. (2023). Detection of Various Microplastics in Placentas, Meconium, Infant Feces, Breastmilk and Infant Formula: A Pilot Prospective Study. Sci. Total Environ..

[16] Amereh F., Amjadi N., Mohseni-Bandpei A., Isazadeh S., Mehrabi Y., Eslami A., Naeiji Z., Rafiee M. (2022). Placental Plastics in Young Women from General Population Correlate with Reduced Foetal Growth in IUGR Pregnancies. Environ. Pollut..

[17] Ragusa A., Principi G., Matta M. (2022). Pregnancy in the Era of the Environmental Crisis: Plastic and Pollution. Clin. Exp. Obstet. Gynecol..

[18] Zhang H., Ding X., Zheng H., Ma Q., Zhang T. (2025). Health Risks of Prenatal and Early-Life Microplastics Exposure: A Comprehensive Review. Environ. Health.

[19] Lee J., Kim H.-B., Jung H.-J., Chung M., Park S.E., Lee K.-H., Kim W.S., Moon J.-H., Lee J.W., Shim J.W. (2024). Protecting Our Future: Environmental Hazards and Children’s Health in the Face of Environmental Threats: A Comprehensive Overview. Clin. Exp. Pediatr..

[20] Botelho R.M., Silva A.L.M., Borbely A.U. (2024). The Autism Spectrum Disorder and Its Possible Origins in Pregnancy. Int. J. Environ. Res. Public Health.

[21] Page M.J., McKenzie J.E., Bossuyt P.M., Boutron I., Hoffmann T.C., Mulrow C.D., Shamseer L., Tetzlaff J.M., Akl E.A., Brennan S.E. (2021). The PRISMA 2020 Statement: An Updated Guideline for Reporting Systematic Reviews. BMJ.

[22] Campbell M., McKenzie J.E., Sowden A., Katikireddi S.V., Brennan S.E., Ellis S., Hartmann-Boyce J., Ryan R., Shepperd S., Thomas J. (2020). Synthesis without Meta-Analysis (SWiM) in Systematic Reviews: Reporting Guideline. BMJ.

[23] Xie R., Xiao X., Zhao W., Zhong Y., Wu D., Dou J., Zhao Y., Luo Y., Cao Y., Chang Y. (2025). Association between Long-Term Exposure of Polystyrene Microplastics and Exacerbation of Seizure Symptoms: Evidence from Multiple Approaches. Ecotoxicol. Environ. Saf..

[24] Moola S., Munn Z., Tufanaru C., Aromataris E., Sears K., Sfetcu R., Currie M., Qureshi R., Mattis P., Lisy K. (2020). Chapter 7: Systematic Reviews of Etiology and Risk. Joanna Briggs Institute Reviewer’s Manual.

[25] Dong L., Li X., Zhang Y., Liu B., Zhang X., Yang L. (2025). Urinary Microplastic Contaminants in Primary School Children: Associations with Behavioral Development. Ecotoxicol. Environ. Saf..

[26] Zheng D., Wang D., Zhang S., Liu Y., Xi Q., Weng Y. (2025). Impact of Urinary Microplastic Exposure on Cognitive Function in Primary School Children. Ecotoxicol. Environ. Saf..

[27] Guyatt G.H., Oxman A.D., Vist G.E., Kunz R., Falck-Ytter Y., Alonso-Coello P., Schünemann H.J. (2008). GRADE: An Emerging Consensus on Rating Quality of Evidence and Strength of Recommendations. BMJ.

[28] Scott A.J., Sharpe L., Loomes M., Gandy M. (2020). Systematic Review and Meta-Analysis of Anxiety and Depression in Youth with Epilepsy. J. Pediatr. Psychol..

[29] Leslie H.A., Van Velzen M.J.M., Brandsma S.H., Vethaak A.D., Garcia-Vallejo J.J., Lamoree M.H. (2022). Discovery and Quantification of Plastic Particle Pollution in Human Blood. Environ. Int..

[30] Yaffe J., Montgomery P., Hopewell S., Shepard L.D. (2012). Empty Reviews: A Description and Consideration of Cochrane Systematic Reviews with No Included Studies. PLoS ONE.

[31] Makwana B., Desai B., Srinivasan J., Apetauerova D., Dani S.S., Sehgal S., Yerstein O., Khadke S., Kumar A., Nasir K. (2025). Impact of Marine Microplastics on Neurologic and Functional Disabilities: A Population-Level Study. Eur. J. Neurol..

[32] Landrigan P.J., Stegeman J.J., Fleming L.E., Allemand D., Anderson D.M., Backer L.C., Brucker-Davis F., Chevalier N., Corra L., Czerucka D. (2020). Human Health and Ocean Pollution. Ann. Glob. Health.

[33] Newson J.J., Marinova Z., Thiagarajan T.C. (2025). Are the Growing Levels of Neurotoxic and Neuro-Disruptive Chemicals in Our Food and Drink Contributing to the Youth Mental Health Crisis? A Narrative Review. Neurosci. Biobehav. Rev..

[34] Schwabl P., Köppel S., Königshofer P., Bucsics T., Trauner M., Reiberger T., Liebmann B. (2019). Detection of Various Microplastics in Human Stool: A Prospective Case Series. Ann. Intern. Med..

[35] Wu P., Lin S., Cao G., Wu J., Jin H., Wang C., Wong M.H., Yang Z., Cai Z. (2022). Absorption, Distribution, Metabolism, Excretion and Toxicity of Microplastics in the Human Body and Health Implications. J. Hazard. Mater..

[36] Miguela-Benavides M., Calikanzaros E., Donat-Vargas C., Aguilar R., Raimondi F., Iraola S., Dobaño C., De Cid R., Llorca M., Farré M. (2026). Association of Faecal and Urinary Micro- and Nanoplastics with Markers of Gut Integrity and Renal Function. Environ. Res..

[37] Dzierżyński E., Blicharz-Grabias E., Komaniecka I., Panek R., Forma A., Gawlik P.J., Puźniak D., Flieger W., Choma A., Suśniak K. (2025). Post-Mortem Evidence of Microplastic Bioaccumulation in Human Organs: Insights from Advanced Imaging and Spectroscopic Analysis. Arch. Toxicol..

[38] Lamoree M.H., Van Boxel J., Nardella F., Houthuijs K.J., Brandsma S.H., Béen F., Van Duursen M.B.M. (2025). Health Impacts of Microplastic and Nanoplastic Exposure. Nat. Med..

[39] Song Y.K., Hong S.H., Eo S., Shim W.J. (2021). A Comparison of Spectroscopic Analysis Methods for Microplastics: Manual, Semi-Automated, and Automated Fourier Transform Infrared and Raman Techniques. Mar. Pollut. Bull..

[40] Sourander A., Westerlund M., Kaneko H., Heinonen E., Klomek A.B., How Ong S., Fossum S., Kolaitis G., Lesinskiene S., Li L. (2025). Cross-Cultural Comparison of the Strengths and Difficulties Self-Report Questionnaire in 12 Asian and European Countries. J. Am. Acad. Child. Adolesc. Psychiatry.

[41] Al-Beltagi M., Saeed N.K. (2022). Epilepsy and the Gut: Perpetrator or Victim?. World J. Gastrointest. Pathophysiol..

[42] Huang K., Tong J., Tao S., Wu X., Yan S., Gao G., Cao H., Xie L., Gao H., Geng M. (2024). Cohort Profile: The Ma’anshan Birth Cohort (MABC) Study. Int. J. Epidemiol..

[43] Sekovanić A., Orct T., Kljaković-Gašpić Z. (2025). Micro- and Nanoplastics and Fetal Health: Challenges in Assessment and Evidence from Epidemiological Studies. Toxics.

[44] Krause K.R., Chung S., Adewuya A.O., Albano A.M., Babins-Wagner R., Birkinshaw L., Brann P., Creswell C., Delaney K., Falissard B. (2021). International Consensus on a Standard Set of Outcome Measures for Child and Youth Anxiety, Depression, Obsessive-Compulsive Disorder, and Post-Traumatic Stress Disorder. Lancet Psychiatry.

[45] Solé D., Camargos P.A.M. (2025). Childhood Health on a Planet Threatened by Climate Change. J. Pediatr..

