# Supplementary material for: Exposure to Microplastics in Biological Matrices and Neurodevelopmental Outcomes in Children: A Systematic Review
Source: Nanomaterials (Basel). 2026 May 18;16(10):618. doi: 10.3390/nano16100618 (PMC13210297; doi:10.3390/nano16100618)
Supplement: Supplementary file 1 [file nanomaterials-16-00618-s001.zip › nanomaterials-4285222-supplementary.pdf]

Supplementary File S1

Exposure to Microplastics in Biological Matrices and Neurodevelopmental Outcomes in Children: A Systematic Review

Francesco Fabrizio Comisi 1,†, Andrea Maria Comisi 2,†, Elena Esposito 1,* and Vassilios Fanos 1

1 Neonatal Intensive Care Unit, Department of Surgical Sciences, University of Cagliari, 09124 Cagliari, Italy

2 Pediatric Unit, Department of Clinical and Experimental Medicine, University of Catania, 95131 Catania, Italy

* Correspondence: elena.esposito30@gmail.com

† These authors contributed equally to this work.

Complete Electronic Search Strategies

All searches were conducted on 19 February 2026. No date restrictions were applied. The search strategy combined three concept groups using Boolean operators: (1) microplastics and nanoplastics; (2) pediatric populations; and (3) neurodevelopmental outcomes. Strategies were adapted to the syntax and controlled vocabulary of each database.

PubMed

```
( microplastic*[tiab] OR nanoplastic*[tiab] OR "micro- and nanoplastic*"[tiab] OR
"plastic particle*"[tiab] )
AND
( child*[tiab] OR pediatric*[tiab] OR paediatric*[tiab] OR infant*[tiab] OR
neonat*[tiab] OR adolescen*[tiab] OR newborn*[tiab] OR toddler*[tiab] OR
"early life"[tiab] )
AND
( neurodevelop*[tiab] OR "neurodevelopmental"[tiab] OR "developmental delay"[tiab] OR
"brain development"[tiab] OR neurolog*[tiab] OR neurotox*[tiab] OR
neuroinflamm*[tiab] OR cognit*[tiab] OR learning[tiab] OR memory[tiab] OR
"executive function"[tiab] OR behavio*[tiab] OR neurobehavio*[tiab] OR autism[tiab] OR
ASD[tiab] OR ADHD[tiab] OR "attention deficit"[tiab] OR seizure*[tiab] OR
epilep*[tiab] OR encephalopath*[tiab] OR "motor development"[tiab] OR
"language development"[tiab] )
AND English[lang]
```

Scopus

```
( TITLE-ABS-KEY ( microplastic* OR nanoplastic* OR "micro- and nanoplastic*" OR
"plastic particle*" ) )
AND
( TITLE-ABS-KEY ( child* OR pediatric* OR paediatric* OR infant* OR neonat* OR
adolescen* OR newborn* OR toddler* OR "early life" ) )
AND
( TITLE-ABS-KEY ( neurodevelop* OR neurodevelopmental OR "developmental delay" OR
"brain development" OR neurolog* OR neurotox* OR neuroinflamm* OR cognit* OR
learning OR memory OR "executive function" OR behavio* OR neurobehavio* OR autism OR
ASD OR ADHD OR "attention deficit" OR seizure* OR epilep* OR encephalopath* OR
"motor development" OR "language development" ) )
```

AND
(LIMIT-TO (LANGUAGE , "English"))

Cochrane Library - CENTRAL

(microplastic* OR nanoplastic* OR "micro- and nanoplastic*" OR "plastic particle*")
AND
(child* OR pediatric* OR paediatric* OR infant* OR neonat* OR adolescen* OR newborn* OR toddler* OR "early life")
AND
(neurodevelop* OR "developmental delay" OR "brain development" OR neurolog* OR neurotox* OR neuroinflamm* OR cognit* OR learning OR memory OR "executive function" OR behavio* OR neurobehavio* OR autism OR ASD OR ADHD OR "attention deficit" OR seizure* OR epilep* OR encephalopath* OR "motor development" OR "language development")

Web of Science

TS=(microplastic* OR nanoplastic* OR "micro- and nanoplastic*" OR "plastic particle*")
AND
TS=(child* OR pediatric* OR paediatric* OR infant* OR neonat* OR adolescen* OR newborn* OR toddler* OR "early life")
AND
TS=(neurodevelop* OR "developmental delay" OR "brain development" OR neurolog* OR neurotox* OR neuroinflamm* OR cognit* OR learning OR memory OR "executive function" OR behavio* OR neurobehavio* OR autism OR ASD OR ADHD OR "attention deficit" OR seizure* OR epilep* OR encephalopath* OR "motor development" OR "language development")

ClinicalTrials.gov

(microplastic OR nanoplastic OR "plastic particles")
AND
(child OR infant OR neonate OR adolescent OR pediatric)
AND
(neurodevelopment OR neurotoxicity OR cognitive OR behavior OR autism OR ADHD)

WHO ICTRP

microplastic OR nanoplastic AND child OR infant AND neurodevelopment OR neurotoxicity

---

Full-Text Articles Excluded with Reasons (n = 18)

Of 21 full-text articles assessed for eligibility, 3 met all inclusion criteria and 18 were excluded. The table below lists each excluded article with its specific exclusion reason, sorted by category.

Ineligible study design (n = 10)

#	Study	Title	Journal
1	Landrigan PJ, 2020	Human Health and Ocean Pollution	Annals of Global Health
2	Antonio Ragusa, 2022	Pregnancy in the Era of the Environmental Crisis: Plastic and Pollution	Clinical and Experimental Obstetrics and Gynecology
3	Symeonides C, 2024	An Umbrella Review of	Annals of Global Health

#	Study	Title	Journal
		Meta-Analyses Evaluating Associations between Human Health Outcomes and Plastic Exposure	
4	Haopeng Zhang, 2025	Health Risks of Prenatal and Early-Life Microplastics Exposure: A Comprehensive Review	Environment and Health
5	Luo Q, 2025	Microplastics as an emerging threat to human health: An overview of potential health risks	Journal of Environmental Management
6	Nadarasan S, 2025	Microplastics and child health: A scoping review of prenatal and early-life exposures	Toxicology Reports
7	Newson JJ, 2025	Are the growing levels of neurotoxic and neuro-disruptive chemicals in our food and environment degrading our mental health?	Neuroscience and Biobehavioral Reviews
8	Ragusa, 2025	Microplastics and nanoplastics in the brain: a review of the neurodevelopmental effects	Journal of Pediatric and Neonatal Individualized Medicine
9	Sekovanić A, 2025	Micro- and Nanoplastics and Fetal Health: Challenges in Assessment and Evidence	Toxics
10	Sole D, 2025	Childhood health on a planet threatened by climate change	Jornal de Pediatria

Note: This category includes narrative reviews, scoping reviews, umbrella reviews, editorials, and commentaries that did not present original observational data.

Exposure not pertinent (n = 4)

#	Study	Title	Journal
11	Botelho RM, 2024	The Autism Spectrum Disorder and Its Possible Origins in Pregnancy	International Journal of Environmental Research and Public Health
12	Dennis, 2025	Unseen toxins: Exploring the human health consequences of micro and nanoplastics	Toxicology Reports
13	Esposito, 2025	The Silent Conquest: The Journey of Micro- and	Toxics

#	Study	Title	Journal
		Nanoplastics Through Children's Organs	
14	Lane T, 2025	Exposure scenarios for human health risk assessment of nano- and microplastic particles	Microplastics and Nanoplastics

Note: These studies did not measure MNP particles in human biological matrices; they addressed chemical additives, exposure modeling, or MNP distribution narratively.

In vitro/animal only (n = 2)

#	Study	Title	Journal
15	Amran NH, 2022	Exposure to Microplastics during Early Developmental Stage: Review of Current Evidence	Toxics
16	Lee J, 2024	Protecting our future: environmental hazards and children's health in the face of climate change	Clinical and Experimental Pediatrics

No neurodevelopmental outcome (n = 2)

#	Study	Title	Journal
17	Huang, 2024	Cohort profile: The Ma'anshan birth cohort (MABC) study	International Journal of Epidemiology
18	Liu S, 2023	Detection of various microplastics in placentas, meconium, infant feces, breastmilk, and infant formula	Science of the Total Environment

Note: Huang 2024 is a pediatric birth cohort profile that describes study infrastructure, including planned MNP assessments, but does not report neurodevelopmental outcome data in relation to MNP exposure. Liu 2023 measured MNPs in pediatric biological matrices but did not assess neurodevelopmental outcomes.

SWiM Reporting Checklist (Campbell et al. [22])

SWiM Item	Requirement	Where/How Addressed
1. Grouping of studies for synthesis	Define groups and present results accordingly	Results grouped by individual study (Section 2.8 and Sections 3.4.1-3.4.3) given three studies with non-overlapping outcome domains (behavioral, cognitive, neurological)
2. Describe the standardized metric and transformation methods	Report the metric used for each synthesis	Adjusted regression coefficients (β coefficients with 95% CI and p-values), unadjusted group comparisons, and Spearman correlations reported as provided by original studies (Tables 2-4); no transformations applied
3. Describe the synthesis methods	Specify the synthesis method for each group	Narrative synthesis with structured tabular presentation; results organized by study and then by exposure-outcome pair
4. Criteria used to prioritize results for summary and synthesis	State which results were prioritized	Adjusted models prioritized over crude where available; Dong: Model 2 (fully adjusted); Zheng: Model 2 (fully adjusted); Xie: unadjusted analyses reported (groups controlled at selection for age, sex, SES, and comorbidities per author correspondence, but no multivariable model in

SWiM Item	Requirement	Where/How Addressed
		reported analyses)
5. Methods to examine heterogeneity	Describe methods used to investigate between-study variation	Formal heterogeneity assessment not applicable: three studies addressed non-overlapping outcome domains with no common measure across studies
6. Certainty of evidence	Describe the method of assessing certainty	GRADE framework applied to each exposure-outcome pair (Section 2.7); all 14 outcomes rated Very Low certainty (Supplementary Table S4)
7. Data presentation methods	Describe how data are presented	Structured tables by study: Table 2 (Zheng, cognitive), Table 3 (Dong, behavioral), Table 4 (Xie, neurological); forest plots not applicable
8. Reporting biases	Describe assessment of reporting biases	Publication bias assessed qualitatively (Section 3.5, paragraphs 4-5; Section 4.5.4); funnel plots not feasible with single-study evidence per outcome; within-study selective reporting assessed for all three studies (28 Dong, 18 Zheng, 10 Xie; 56 associations total); multiple comparison concern noted for Dong and Zheng (Section 4.5.2)
9. Deviations from the protocol	Report any deviations from the registered protocol	Section 2.1: "No protocol deviations occurred"; pre-specified outcome domains (e.g., prenatal MNP exposure) were not identified in eligible studies

PRISMA 2020 Checklist (Page et al. [21])

Section and Topic	Item #	Checklist Item	Location Where Reported
**TITLE**			
Title	1	Identify the report as a systematic review.	Title (line 1): "…A Systematic Review"
**ABSTRACT**			
Abstract	2	See the PRISMA 2020 for Abstracts checklist (below).	Abstract
**INTRODUCTION**			
Rationale	3	Describe the rationale for the review in the context of existing knowledge.	Section 1 (Introduction), paragraphs 1-3: mechanistic plausibility, gap in prior reviews (Symeonides 2024, Vojnits 2025, Nadarasan 2025)
Objectives	4	Provide an explicit statement of the objective(s) or question(s) the review addresses.	Section 1 (Introduction), final paragraph: PECOS framework and primary objective stated
**METHODS**			
Eligibility criteria	5	Specify the inclusion and exclusion criteria for the review and how studies were grouped for the syntheses.	Section 2.2 (Eligibility Criteria): PECOS criteria, exclusion criteria; Section 2.8 (Data Synthesis): grouping rationale
Information sources	6	Specify all databases, registers, websites, organisations, reference lists and other sources searched or consulted to identify studies. Specify the date when each source was last searched or consulted.	Section 2.3 (Information Sources and Search Strategy): six databases searched on 19 February 2026; forward and backward citation searching described
Search strategy	7	Present the full search strategies for all databases, registers and websites, including any filters and limits used.	Section 2.3 references Supplementary File S1; Supplementary File S1, Section "Complete Electronic Search Strategies": full strategies for all six databases
Selection process	8	Specify the methods used to decide whether a study met the inclusion criteria of the review, including how many reviewers screened each record and each report retrieved, whether they worked independently, and if applicable, details of automation tools used in the process.	Section 2.4 (Study Selection): two independent reviewers (A.M.C., F.F.C.), third-reviewer arbitration (E.E.), Cohen's kappa reported; Section 2.5: no automation tools used
Data collection process	9	Specify the methods used to collect data from reports, including how many reviewers collected data from each report, whether they worked independently, any processes for obtaining or confirming data from study investigators, and if applicable,	Section 2.5 (Data Extraction): two independent reviewers, standardized form, discrepancy rate (13.3%), consensus with third-reviewer arbitration, authors of Dong and Zheng contacted (no response); corresponding author of Xie

Section and Topic	Item #	Checklist Item	Location Where Reported
		details of automation tools used in the process.	responded with supplementary data (see Section 2.5), no automation tools
Data items	10a	List and define all outcomes for which data were sought. Specify whether all results that were compatible with each outcome domain in each study were sought (e.g. for all measures, time points, analyses), and if not, the methods used to decide which results to collect.	Section 2.2 (Eligibility Criteria): outcomes listed; Section 2.1 (Protocol and Registration): pre-specified outcome domains identified; Section 3.5 (Certainty of Evidence), paragraph 4: selective outcome reporting assessment
	10b	List and define all other variables for which data were sought (e.g. participant and intervention characteristics, funding sources). List any assumptions made about any missing or unclear information.	Section 2.5 (Data Extraction): standardized form; Table 1 (Study Characteristics): variables extracted (design, N, age, location, matrix, method, outcome); Section 3.2: funding sources reported
Study risk of bias assessment	11	Specify the methods used to assess risk of bias in the included studies, including details of the tool(s) used, how many reviewers assessed each study and whether they worked independently, and if applicable, details of automation tools used in the process.	Section 2.6 (Risk of Bias Assessment): JBI checklists specified (cross-sectional 8 items, case-control 10 items), two reviewers, Cohen's kappa = 0.843
Effect measures	12	Specify for each outcome the effect measure(s) (e.g. risk ratio, mean difference) used in the synthesis or presentation of results.	Section 2.2 (Eligibility Criteria): effect measures listed (regression coefficients, odds ratios, correlation coefficients, mean differences with 95% CI); Tables 2-4: specific measures per study
Synthesis methods	13a	Describe the processes used to decide which studies were eligible for each synthesis (e.g. tabulating the study intervention characteristics and comparing against the planned groups for each synthesis).	Section 2.8 (Data Synthesis): three reasons meta-analysis was not performed; studies grouped by individual study given non-overlapping outcome domains
	13b	Describe any methods required to prepare the data for presentation or synthesis, such as handling of missing summary statistics, or data conversions.	Tables 2-4 footnotes: effect estimates reported as provided by original studies; no transformations applied; Supplementary File S1, SWiM Item 2
	13c	Describe any methods used to tabulate or visually display results of individual studies and syntheses.	Tables 2-4: structured by study; Supplementary Table S3 (Summary of Findings); Supplementary Table S4 (Evidence Profile)
	13d	Describe any methods used to synthesize results and provide a rationale for the choice(s). If meta-analysis was performed, describe the model(s), method(s) to identify the presence and extent of statistical heterogeneity, and software package(s) used.	Section 2.8 (Data Synthesis): narrative synthesis following SWiM (Campbell et al. 2020); meta-analysis not performed (rationale given)
	13e	Describe any methods used to explore possible causes of heterogeneity among study results (e.g. subgroup analysis, meta-regression).	Section 2.8 and Supplementary File S1, SWiM Item 5: formal heterogeneity assessment not applicable (non-overlapping outcome domains)
	13f	Describe any sensitivity analyses conducted to assess robustness of the synthesized results.	Supplementary File S1, SWiM Item 8: sensitivity analyses not performed (insufficient studies)
Reporting bias assessment	14	Describe any methods used to assess risk of bias due to missing results in a synthesis (arising from reporting biases).	Section 2.8 (Data Synthesis): qualitative assessment described; Supplementary File S1, SWiM Item 8: reporting bias methods; Section 3.5, paragraphs 4-5: selective outcome reporting assessed per study; Section 4.5.4 (Generalizability and Reporting): publication bias considerations

Section and Topic	Item #	Checklist Item	Location Where Reported
Certainty assessment	15	Describe any methods used to assess certainty (or confidence) in the body of evidence for an outcome.	Section 2.7 (Certainty of Evidence): GRADE approach described, starting at Low for observational evidence, five downgrading and three upgrading domains evaluated
**RESULTS**			
Study selection	16a	Describe the results of the search and selection process, from the number of records identified in the search to the number of studies included in the review, ideally using a flow diagram.	Section 3.1 (Study Selection): numbers at each stage; Figure 1 (PRISMA 2020 flow diagram)
	16b	Cite studies that might appear to meet the inclusion criteria, but which were excluded, and explain why they were excluded.	Section 3.1 references Supplementary File S1; Supplementary File S1, Section "Full-Text Articles Excluded with Reasons (n = 18)": all 18 articles listed individually with reasons
Study characteristics	17	Cite each included study and present its characteristics.	Section 3.2 (Study Characteristics): narrative description; Table 1 (Characteristics of Included Studies)
Risk of bias in studies	18	Present assessments of risk of bias for each included study.	Section 3.3 (Risk of Bias Assessment): narrative; Supplementary Tables S1 and S2 (JBI domain-level ratings for each study)
Results of individual studies	19	For all outcomes, present, for each study: (a) summary statistics for each group (where appropriate) and (b) an effect estimate and its precision (e.g. confidence/credible interval), ideally using structured tables or forest plots.	Section 3.4 (Individual Study Results): Sections 3.4.1-3.4.3 narrative; Tables 2-4 (effect estimates with 95% CI and p-values)
Results of syntheses	20a	For each synthesis, briefly summarise the characteristics and risk of bias among contributing studies.	Section 3.5 (Certainty of Evidence): risk of bias summarized per study group; Supplementary Table S4 (Evidence Profile): RoB column
	20b	Present results of all statistical syntheses conducted. If meta-analysis was done, present for each the summary estimate and its precision (e.g. confidence/credible interval) and measures of statistical heterogeneity. If comparing groups, describe the direction of the effect.	N/A, meta-analysis not performed; narrative synthesis results in Sections 3.4.1-3.4.3 with direction of effect described
	20c	Present results of all investigations of possible causes of heterogeneity among study results.	N/A, heterogeneity investigation not applicable (non-overlapping outcome domains; Section 2.8)
	20d	Present results of all sensitivity analyses conducted to assess the robustness of the synthesized results.	N/A, sensitivity analyses not performed (Supplementary File S1, SWiM Item 8)
Reporting biases	21	Present assessments of risk of bias due to missing results (arising from reporting biases) for each synthesis.	Section 3.5, paragraphs 4-5: selective outcome reporting per study; Section 4.5.4 (Generalizability and Reporting): publication bias discussion
Certainty of evidence	22	Present assessments of certainty (or confidence) in the body of evidence for each outcome assessed.	Section 3.5 (Certainty of Evidence): narrative; Supplementary Table S3 (Summary of Findings); Supplementary Table S4 (Evidence Profile with GRADE ratings)
**DISCUSSION**			
Discussion	23a	Provide a general interpretation of the results in the context of other evidence.	Section 4.1 (Summary and Comparison with Existing Literature); Section 4.2 (Environmental Analogy); Section 4.3 (Biological Plausibility)
	23b	Discuss any limitations of the evidence included in the review.	Section 4.5 (Limitations): four subsections (4.5.1 Exposure assessment; 4.5.2 Confounding and causal inference; 4.5.3 Outcome assessment; 4.5.4 Generalizability and

Section and Topic	Item #	Checklist Item	Location Where Reported
			reporting)
	23c	Discuss any limitations of the review processes used.	Section 4.4 (Strengths): retrospective registration; Section 4.5.4 (Generalizability and reporting): English-language restriction, urban China sampling, probable population overlap between Dong and Zheng (also Section 3.2); author non-response for Dong and Zheng and Xie author correspondence are reported in Section 2.5
	23d	Discuss implications of the results for practice, policy, and future research.	Section 4.6 (Implications): three subsections (For research, For clinical practice, For policy)
**OTHER INFORMATION**			
Registration and protocol	24a	Provide registration information for the review, including register name and registration number, or state that the review was not registered.	Section 2.1 (Protocol and Registration): PROSPERO CRD420261328979; retrospective registration disclosed; protocol deviations stated
	24b	Indicate where the review protocol can be accessed, or state that a protocol was not prepared.	Section 2.1: registered on PROSPERO (protocol accessible via PROSPERO registry)
	24c	Describe and explain any amendments to information provided at registration or in the protocol.	Section 2.1: “No protocol deviations occurred”; pre-specified outcomes not found in eligible studies noted
Support	25	Describe sources of financial or non-financial support for the review, and the role of the funders or sponsors in the review.	Funding section: “This research received no external funding”
Competing interests	26	Declare any competing interests of review authors.	Conflicts of Interest section: “The authors declare no conflicts of interest”
Availability of data, code, and other materials	27	Specify which of the following are publicly available and where they can be found: template data collection forms; data extracted from included studies; data used for all analyses; analytic code; any other materials used in the review.	Data Availability Statement: “All data supporting the findings of this systematic review are contained within the article. No new data were created or analyzed in this study.”

PRISMA 2020 for Abstracts Checklist (Page et al. [21])

Section and Topic	Item #	Checklist Item	Location Where Reported
**TITLE**			
Title	1	Identify the report as a systematic review.	Title: “…A Systematic Review”
**ABSTRACT**			
Objectives	2	Provide an explicit statement of the main objective(s) or question(s) the review addresses.	Abstract, aim sentence: “We aimed to systematically identify, appraise, and synthesize observational evidence on this association in children aged 0-18 years”
Eligibility criteria	3	Specify the inclusion criteria for the review.	Abstract, aim sentence: “observational evidence on this association in children aged 0-18 years”; full eligibility (PECOS) reported in Section 2.2
Information sources	4	Specify the information sources (e.g. databases, registers) used to identify studies and the date when each was last searched.	Abstract, Methods: “Six databases were searched on 19 February 2026”
Risk of bias	5	Specify the methods used to assess risk of bias in the included studies.	Abstract, Methods: “Risk of bias and certainty of evidence were assessed using JBI checklists and the GRADE framework, respectively”

Section and Topic	Item #	Checklist Item	Location Where Reported
Synthesis of results	6	Specify the methods used to present and synthesise results.	Abstract, Methods: "systematically identified, appraised, and synthesized" (narrative synthesis implied; PRISMA 2020 and PROSPERO cited)
Results, Included studies	7	Give the total number of included studies and participants and summarise relevant characteristics of studies.	Abstract, Results: "Three studies met the inclusion criteria (all published in 2025, China; n = 30-5670; two studies with probable population overlap)"
Results, Synthesis of results	8	Present results for main outcomes, preferably indicating the number of included studies and participants for each. If meta-analysis was done, report the summary estimate and confidence/credible interval. If comparing groups, indicate the direction of the effect.	Abstract, Results: "addressing behavioral, cognitive, and neurological outcome domains, encompassing 56 associations across 14 outcomes. Each study showed a uniform direction of association (higher MP exposure was associated with poorer outcomes); however, probable population overlap between Dong and Zheng precludes interpretation of this pattern as independent cross-study replication. All outcomes were rated Very Low certainty under GRADE; meta-analysis was not performed."
Limitations of evidence	9	Provide a brief summary of the limitations of the evidence included in the review (e.g. study risk of bias, inconsistency and imprecision).	Abstract, Results: "All outcomes were rated Very Low certainty under GRADE"; Abstract, Conclusions: "no causal inferences can be drawn in the absence of independent replication, and the field remains at the stage of hypothesis generation"
Interpretation	10	Provide a general interpretation of the results and important implications.	Abstract, Conclusions: consistency of associations, biological plausibility, relevance as research direction
Funding	11	Specify the primary source of funding for the review.	Not stated in abstract. Reported in Funding section of manuscript: "This research received no external funding"
Registration	12	Provide the register name and registration number.	Abstract, Methods: "PROSPERO: CRD420261328979"

Supplementary Tables

Supplementary Table S1. Risk of Bias Assessment: JBI Checklist for Analytical Cross-Sectional Studies

JBI Domain	Zheng (2025)	Dong (2025)
1. Inclusion criteria clearly defined	Y	Y
2. Subjects and setting described in detail	Y	Y
3. Exposure measured validly and reliably	N	N
4. Objective criteria for condition measurement	Y	Y
5. Confounding factors identified	Y	Y
6. Strategies for confounders stated	Y	Y
7. Outcomes measured validly and reliably	Y	Y
8. Appropriate statistical analysis	U	Y

Abbreviations: JBI, Joanna Briggs Institute. Y = Yes; N = No; U = Unclear; NA = Not applicable.

Supplementary Table S2. Risk of Bias Assessment: JBI Checklist for Case-Control Studies

JBI Domain	Xie (2025)
1. Groups comparable other than disease presence/absence	U
2. Cases and controls matched appropriately	Y
3. Same criteria for identification of cases and controls	U
4. Exposure measured in a standard, valid and reliable way	Y

JBI Domain	Xie (2025)
5. Exposure measured in the same way for cases and controls	Y
6. Confounding factors identified	N
7. Strategies to deal with confounding factors stated	N
8. Outcomes assessed in a standard, valid and reliable way	Y
9. Exposure period of interest long enough to be meaningful	N
10. Appropriate statistical analysis used	N

Abbreviations: JBI, Joanna Briggs Institute. Y = Yes; N = No; U = Unclear; NA = Not applicable.

Supplementary Table S3. Summary of Findings: GRADE certainty of evidence for associations between total microplastic exposure and neurodevelopmental outcomes.

Outcome	Study	Design	N	Effect estimate (Total MPs)	Certainty of evidence
Working memory (Two-Back d’)	Zheng 2025	Cross-sectional	5670	beta = -7.42 (95% CI: -10.27 to -4.56; p < 0.001)	Very Low (⊕◯◯◯)
Superior working memory (Three-Back d’)	Zheng 2025	Cross-sectional	5670	beta = -2.25 (95% CI: -3.34 to -1.16; p < 0.001)	Very Low (⊕◯◯◯)
Inattentiveness (HRT-SE)	Zheng 2025	Cross-sectional	5670	beta = -1.83 (95% CI: -3.57 to -0.09; p = 0.04)	Very Low (⊕◯◯◯)
Emotional problems (SDQ)	Dong 2025	Cross-sectional	1000	beta = 0.128 (95% CI: 0.062 to 0.195; p < 0.001)	Very Low (⊕◯◯◯)
Conduct problems (SDQ)	Dong 2025	Cross-sectional	1000	beta = 0.209 (95% CI: 0.146 to 0.273; p < 0.001)	Very Low (⊕◯◯◯)
Hyperactivity-inattention (SDQ)	Dong 2025	Cross-sectional	1000	beta = 0.168 (95% CI: 0.101 to 0.235; p < 0.001)	Very Low (⊕◯◯◯)
Peer relationship problems (SDQ)	Dong 2025	Cross-sectional	1000	beta = 0.206 (95% CI: 0.137 to 0.275; p < 0.001)	Very Low (⊕◯◯◯)
Prosocial behavior (SDQ)	Dong 2025	Cross-sectional	1000	beta = -0.125 (95% CI: -0.196 to -0.055; p = 0.001)	Very Low (⊕◯◯◯)
Total difficulties score (SDQ)	Dong 2025	Cross-sectional	1000	beta = 0.177 (95% CI: 0.148 to 0.206; p < 0.001)	Very Low (⊕◯◯◯)
Impact supplement (SDQ)	Dong 2025	Cross-sectional	1000	beta = -0.022 (95% CI: -0.092 to 0.047; p = 0.528)	Very Low (⊕◯◯◯)
Seizure severity	Xie 2025	Case-control	30	Higher MP levels in seizure group vs controls (p < 0.05)	Very Low (⊕◯◯◯)
GAD-7 anxiety	Xie 2025	Case-control	30	Higher in cases (p < 0.05)	Very Low (⊕◯◯◯)
Brain iron (hippocampus)	Xie 2025	Case-control	30	Higher in cases (p < 0.05)	Very Low (⊕◯◯◯)
Blood IL-6	Xie 2025	Case-control	30	R = 0.52 (95% CI: 0.20 to 0.74; p < 0.05)	Very Low (⊕◯◯◯)

Abbreviations: CI, confidence interval; d’, d-prime (signal detection sensitivity index); GAD-7, Generalized Anxiety Disorder 7-item scale; HRT-SE, hit reaction time standard error; IL-6, interleukin-6; MPs, microplastics; R, Spearman correlation coefficient; SDQ, Strengths and Difficulties Questionnaire.

Supplementary Table S4. Evidence profile: GRADE certainty of evidence for all 14 exposure-outcome pairs.

Outcome	Study	No. of studies (N)	Risk of Bias	Inconsistency	Imprecision	Indirectness	Publication Bias	Downgrade Pattern	Overall Certainty
Working memory (Two-Back d’)	Zheng 2025	1 (5670)	Serious (-1)	Cannot assess (0)	Not serious (0)	Serious (-1)	Not serious (0)	RoB (-1) + Indirect (-1)	Very Low (⊕◯◯◯)
Superior working memory (Three-Back d’)	Zheng 2025	1 (5670)	Serious (-1)	Cannot assess (0)	Not serious (0)	Serious (-1)	Not serious (0)	RoB (-1) + Indirect (-1)	Very Low (⊕◯◯◯)
Inattentiveness	Zheng	1 (5670)	Serious (-1)	Cannot assess (0)	Not serious (0)	Serious (-1)	Not serious (0)	RoB (-1) + Indirect (-1)	Very Low (⊕◯◯◯)

Outcome	Study	No. of studies (N)	Risk of Bias	Inconsistency	Imprecision	Indirectness	Publication Bias	Downgrade Pattern	Overall Certainty
(HRT-SE)	2025								
Emotional problems (SDQ)	Dong 2025	1 (1000)	Serious (-1)	Cannot assess (0)	Not serious (0)	Serious (-1)	Not serious (0)	RoB (-1) + Indirect (-1)	Very Low (⊕◯◯◯)
Conduct problems (SDQ)	Dong 2025	1 (1000)	Serious (-1)	Cannot assess (0)	Not serious (0)	Serious (-1)	Not serious (0)	RoB (-1) + Indirect (-1)	Very Low (⊕◯◯◯)
Hyperactivity-inattention (SDQ)	Dong 2025	1 (1000)	Serious (-1)	Cannot assess (0)	Not serious (0)	Serious (-1)	Not serious (0)	RoB (-1) + Indirect (-1)	Very Low (⊕◯◯◯)
Peer relationship problems (SDQ)	Dong 2025	1 (1000)	Serious (-1)	Cannot assess (0)	Not serious (0)	Serious (-1)	Not serious (0)	RoB (-1) + Indirect (-1)	Very Low (⊕◯◯◯)
Prosocial behavior (SDQ)	Dong 2025	1 (1000)	Serious (-1)	Cannot assess (0)	Not serious (0)	Serious (-1)	Not serious (0)	RoB (-1) + Indirect (-1)	Very Low (⊕◯◯◯)
Total difficulties score (SDQ)	Dong 2025	1 (1000)	Serious (-1)	Cannot assess (0)	Not serious (0)	Serious (-1)	Not serious (0)	RoB (-1) + Indirect (-1)	Very Low (⊕◯◯◯)
Impact supplement (SDQ)	Dong 2025	1 (1000)	Serious (-1)	Cannot assess (0)	Not serious (0)	Serious (-1)	Not serious (0)	RoB (-1) + Indirect (-1)	Very Low (⊕◯◯◯)
Seizure severity	Xie 2025	1 (30)	Very serious (-2)	Cannot assess (0)	Very serious (-2)	Serious (-1)	Not serious (0)	RoB (-2) + Imprecision (-2) + Indirect (-1)	Very Low (⊕◯◯◯)
GAD-7 anxiety	Xie 2025	1 (30)	Very serious (-2)	Cannot assess (0)	Very serious (-2)	Very serious (-2)	Not serious (0)	RoB (-2) + Imprecision (-2) + Indirect (-2)	Very Low (⊕◯◯◯)
Brain iron (hippocampus)	Xie 2025	1 (30)	Very serious (-2)	Cannot assess (0)	Very serious (-2)	Serious (-1)	Not serious (0)	RoB (-2) + Imprecision (-2) + Indirect (-1)	Very Low (⊕◯◯◯)
Blood IL-6	Xie 2025	1 (30)	Very serious (-2)	Cannot assess (0)	Very serious (-2)	Serious (-1)	Not serious (0)	RoB (-2) + Imprecision (-2) + Indirect (-1)	Very Low (⊕◯◯◯)

Abbreviations: CI, confidence interval; d’, d-prime (signal detection sensitivity index); GAD-7, Generalized Anxiety Disorder 7-item scale; HRT-SE, hit reaction time standard error; IL-6, interleukin-6; MPs, microplastics; RoB, risk of bias; SDQ, Strengths and Difficulties Questionnaire.

Explanatory footnotes for downgrading decisions:

Risk of bias. Dong and Zheng (Serious, -1): reliance on optical microscopy for urinary MP quantification without systematic spectroscopic verification of all counted particles. Xie (Very serious, -2): no multivariable confounder adjustment in published analyses, single-timepoint fecal exposure measurement, no multiple comparison correction, and bivariate analyses in a sample of 30 participants.

Inconsistency. Cannot assess for all outcomes: single-study evidence per outcome precludes assessment of between-study heterogeneity.

Imprecision. Dong and Zheng (Not serious): adequate sample sizes with available confidence intervals. Xie (Very serious, -2): n = 30 (15 per group) with wide confidence intervals.

Indirectness. Dong and Zheng (Serious, -1): uncertain validity of urinary MPs as a biomarker of systemic MNP burden. Xie (Serious to Very serious, -1 to -2): fecal MPs as indirect marker of systemic exposure; GAD-7 anxiety received -2 owing to the additional concern of using an adult-validated instrument in children without established pediatric validation.

Publication bias. Not serious for all outcomes: formal assessment not feasible with single-study evidence per outcome.
